# Mitochondria-Targeted Antioxidants MitoQ and MitoTEMPO Do Not Influence BRAF-Driven Malignant Melanoma and KRAS-Driven Lung Cancer Progression in Mice

**DOI:** 10.3390/antiox10020163

**Published:** 2021-01-22

**Authors:** Kristell Le Gal, Clotilde Wiel, Mohamed X. Ibrahim, Marcus Henricsson, Volkan I. Sayin, Martin O. Bergo

**Affiliations:** 1 Sahlgrenska Center for Cancer Research, Department of Surgery, Institute of Clinical Sciences, University of Gothenburg, 405 30 Gothenburg, Sweden; kristell.le.gal.beneroso@gu.se (K.L.G.); clotilde.wiel@gu.se (C.W.); volkan.sayin@gu.se (V.I.S.); 2 Wallenberg Centre for Molecular and Translational Medicine, University of Gothenburg, 405 30 Gothenburg, Sweden; 3 Department of Biosciences and Nutrition, Karolinska Institutet, 123 43 Huddinge, Sweden; mohamed.ibrahim@gu.se; 4 Wallenberg laboratory, Institute of Medicine, University of Gothenburg, 405 30 Gothenburg, Sweden; marcus.henricsson@wlab.gu.se

**Keywords:** mitochondria-targeted antioxidants, melanoma, lung cancer, mouse models

## Abstract

Cancer cells produce high levels of mitochondria-associated reactive oxygen species (ROS) that can damage macromolecules, but also promote cell signaling and proliferation. Therefore, mitochondria-targeted antioxidants have been suggested to be useful in anti-cancer therapy, but no studies have convincingly addressed this question. Here, we administered the mitochondria-targeted antioxidants MitoQ and MitoTEMPO to mice with BRAF-induced malignant melanoma and KRAS-induced lung cancer, and found that these compounds had no impact on the number of primary tumors and metastases; and did not influence mitochondrial and nuclear DNA damage levels. Moreover, MitoQ and MitoTEMPO did not influence proliferation of human melanoma and lung cancer cell lines. MitoQ and its control substance dTPP, but not MitoTEMPO, increased glycolytic rates and reduced respiration in melanoma cells; whereas only dTPP produced this effect in lung cancer cells. Our results do not support the use of mitochondria-targeted antioxidants for anti-cancer monotherapy, at least not in malignant melanoma and lung cancer.

## 1. Introduction

Reactive oxygen species (ROS) damage cellular structures and cause oxidative stress, but they also function as signaling molecules that regulate biological and pathological processes [1]. Mitochondria produce a substantial portion of cellular ROS, in particular superoxide (O_2_^●^), as a byproduct of oxidative phosphorylation in complexes I, II, and III of the electron transport chain (ETC) [2]. O_2_^●^ is rapidly converted to hydrogen peroxide (H_2_O_2_) by the enzymatic activity of superoxide dismutases (SODs) located in the mitochondrial matrix [3]. The mitochondria-associated H_2_O_2_ can in turn stimulate intracellular signaling pathways by reversibly oxidizing cysteine residues in key proteins [4,5].

Cancer cells produce high levels of mitochondria-associated H_2_O_2_ that can promote growth and proliferation [6,7]. Indeed, oxidation-induced inactivation of the tumor suppressor protein PTEN increases PI3K signaling and stimulates cell growth and proliferation [8]. Mitochondrial superoxide production can also drive metastasis by activating SRC/PYK2 signaling [9]. Moreover, mutations in genes involved in the ETC, which result in increased production of mitochondrial ROS, have been found in several forms of cancer [10,11].

Consequently, it has been hypothesized that tumor cells can use increased mitochondrial ROS production to their advantage [12]. This idea prompted the development of mitochondria-targeted antioxidants, some of which have produced promising results [9,13,14,15]. However, few studies have addressed the impact of these antioxidants on tumor growth in vivo; to our knowledge, no studies have evaluated their impact on tumor progression in endogenous mouse models of cancer, where physiologic levels of oncogenes initiate tumors in mice with an intact immune system.

In the current study, we administered two well-studied mitochondria-targeted antioxidants, MitoQ and MitoTEMPO, to mice with BRAF-induced malignant melanoma and KRAS-induced lung cancer, and defined their impact on tumor progression and metastasis.

## 2. Materials and Methods

### 2.1. Mice and In Vivo Experiments

For the melanoma model, two-day-old *Braf^CA/+^Pten^fl/fl^ Tyr-Cre^+/0^* mice (designated *BPT*) were painted with 4-hydroxytamoxifen (H6278, Sigma-Aldrich, Darmstadt, Germany) on the right flank skin to activate BRAF^600E^ expression and inactivate PTEN expression in melanocytes, as described [16]. At 3 weeks of age, small nevi were visible on the skin, and littermate mice were randomized to cages with regular drinking water (Ctrl), water supplemented with MitoQ (500 µM), or water supplemented with the control compound decyl-triphenylphosphonium (dTPP, 500 µM) (MitoQ and dTPP were generously provided by Dr. M. Murphy). MitoTEMPO (SML0737, Sigma-Aldrich, Darmstadt, Germany; 1.25 mg/kg body weight in 200 µL PBS) was injected intraperitoneally every two days; control mice were injected with 200 µL PBS. Mice were killed when they became listless because of primary tumor burden, lost 10% of their weight, or when primary tumors ulcerated. *BPT* mice at autopsy exhibit primary tumors and distinct black lymph node metastases [16]. For the lung cancer model, six- to eight-week-old *Kras2^LSL/+^* mice inhaled a *Cre*-adenovirus (5 × 10^7^ plaque-forming units (pfu)) under general anesthesia, as described [17]. One week later, mice were randomized to receive MitoQ- or dTPP-containing water, or regular water; or injected with MitoTEMPO or PBS as outlined above. Mice with lung cancer were killed 10 weeks following Cre-adenovirus inhalation. MitoQ and dTPP administration did not influence body weight and water intake; neither did PBS and MitoTEMPO injections. Mice were on a C57BL/6 genetic background. Mouse experiments were approved by the Research Animal Ethics Committee in Gothenburg (#51-2015 and #52-2015).

### 2.2. Histology

For routine histology, primary tumors and lymph node metastases from *BPT* mice were fixed in paraformaldehyde and embedded in paraffin, and 5-µm sections were stained with hematoxylin and eosin. For lung histology, 4-µm sections of paraformaldehyde inflation– fixed and paraffin-embedded lungs were stained with hematoxylin and eosin. Immunohistochemical analyses were performed as described [16,17]. The sections were incubated with antibodies recognizing Ki67 (RTU (ready-to-use), RM-9106-R7, Thermo Scientific), γ-H2AX (1:1000, ab11174, Abcam, Cambridge, UK), and 8-hydroxyguanosine (1:1000, 48508, Abcam, Cambridge, UK), and then processed with the Vectastain Elite ABC Kit (PK6101) and the DAB Peroxidase Substrate Kit (SK4100, Vector Laboratories). Histological slides were scanned with a MIRAX SCAN microscope and processed with the MIRAX Control software (Zeiss). The scans were quantified with Visiopharm software (Visiopharm Integrator System version 2017.2.5.3857).

### 2.3. Mass Spectrometry

Liver and lung samples were dissected, weighed, snap-frozen, and stored at −80 °C. The content of MitoQ in the tissues was then quantified by liquid chromatography–tandem mass spectrometry (LC/MS/MS) as described [18].

### 2.4. Mitochondrial DNA Damage

Mitochondrial DNA damage was assessed by PCR as described [19]; with minor modifications. Genomic DNA, total RNA, and protein were extracted from tissues preserved with AllProtect Tissue Reagent (76405, Qiagen, Hilden, Germany) using the AllPrep DNA/RNA/protein mini kit (80004, Qiagen, Hilden, Germany) in a QiaCube machine. Up to 30 mg of tissue pieces from primary tumors and lymph node metastases were lysed in RLT buffer in a TissueLyser II (2 × 2-min rounds at 20 Hz). The lysate was centrifuged, and the supernatant transferred into a new 2-mL tube before further processing in the QiaCube. The resulting DNA solution was further cleaned with the Genomic DNA Clean & Concentrator (D4064, Zymo Research, Irvine, CA, USA), and the purity and concentration were assessed by Nanodrop analyses. The DNA was diluted to a concentration of 3 ng/µL, and 5 µL were used per amplification reaction along with 400 nM primer mix and 45 µL Platinum PCR SuperMix, High Fidelity (12532016, Thermo Fisher, Waltham, MA, USA) in a final volume of 54 μL. Duplicates were run for each sample tested along with non-template control samples. The concentrations of amplicons were quantified with Quant-iT PicoGreen dsDNA Assay Kit (P11496, Thermo Fisher, Waltham, MA, USA).

### 2.5. Cell Culture and Proliferation

Human malignant melanoma cell lines (A375 from the American Type Culture Collection; IPC-298 from the German Collection of Microorganisms and Cell Culture) were cultured in DMEM GlutaMAX High Glucose (4.5 g/L, 10569-010) supplemented with 10% fetal bovine serum (10270-106) and 1% penicillin/streptomycin (15070-063). Human lung cancer cell lines A549 and H838 were from the American Type Culture Collection and were cultured in DMEM GlutaMAX Low Glucose (1 g/L, 21885-108) supplemented with 10% fetal bovine serum (10270-106) and 1% penicillin/streptomycin (15070-063, Thermo Fisher, Waltham, MA, USA).

Antioxidant doses were selected based on results from viability assays, which were carried out by seeding 2 × 10^4^ cells per well on 96-well plates. MitoQ, dTPP, and MitoTEMPO (SML0737, Sigma-Aldrich, Darmstadt, Germany) were added at 25, 50, 100, 250, 500, and 1000 nM. Viability was determined 6, 24, and 48 h later using PrestoBlue (A13262, Thermo Fisher, Waltham, MA, USA). The highest concentration that did not affect the viability of untransformed control cells (melanocytes and fibroblasts) was selected for subsequent experiments; this concentration was 100 nM. Real-time analysis of proliferation was assessed by seeding 10^4^ cells per well in 96-well plates in 8 technical replicates each for control medium and medium supplemented with MitoQ, dTPP, or MitoTEMPO. Photomicrographs were taken every 2 h using an IncuCyte Zoom live cell imaging system (Essen Biosciences, Ann Arbor, MI, USA), and cell confluence was measured using the IncuCyte software (Essen Biosciences, Ann Arbor, MI, USA) over 72 h.

### 2.6. Metabolic Flux Analysis

The oxygen consumption rate (OCR) and extra-cellular acidification rate (ECAR) were measured in adherent cells with a XF96 Extracellular Flux Analyzer (Seahorse Bioscience, Agilent, Billerica, MA, USA) using the Seahorse XFCell MitoStress Kit (103015100, Seahorse Agilent, Billerica, MA, USA). Cells were seeded (6–8 replicates) in XF 96-well cell culture microplates (Seahorse Bioscience, Billerica, MA, USA) at a density of 2 × 10^4^ cells/well in 200 μL of DMEM and incubated with MitoQ, dTPP, or MitoTEMPO for 16 h. The medium was replaced with 180 μL 37 °C pre-warmed bicarbonate-free Seahorse XF Base Medium (102353-100, Seahorse Agilent) supplemented with Glucose (5 or 10 mM for lung cancer and melanoma cells, respectively), 1 mM Glutamine, and 1 mM pyruvate. The cells were then incubated for 45 min before starting the assay procedure. After baseline measurements of OCR and ECAR, OCR was again measured after sequentially adding to each well oligomycin and carbonyl cyanide 4-(trifluoromethoxy) phenylhydrazone (FCCP), to reach concentrations of 1 μM. Rotenone/Antimycin A was added to reach a concentration of 0.5 µM. Data were normalized to numbers of viable cells obtained from additional wells using Presto Blue Cell viability. Basal respiration, proton leak, maximal respiration, and spare respiratory capacity were calculated using the MitoStress Test Report Generator.

### 2.7. Statistics

Values are presented as means ± standard error of the mean. GraphPad Prism software (versions 7.02 and 8.1, α < 0.05; San Diego, CA, USA) was used for statistical analyses. One-way ANOVA was used for in vivo analyses. For in vitro studies, one-way ANOVA was used except for cell proliferation, where two-way ANOVA was applied.

## 3. Results

**Figure 1:**
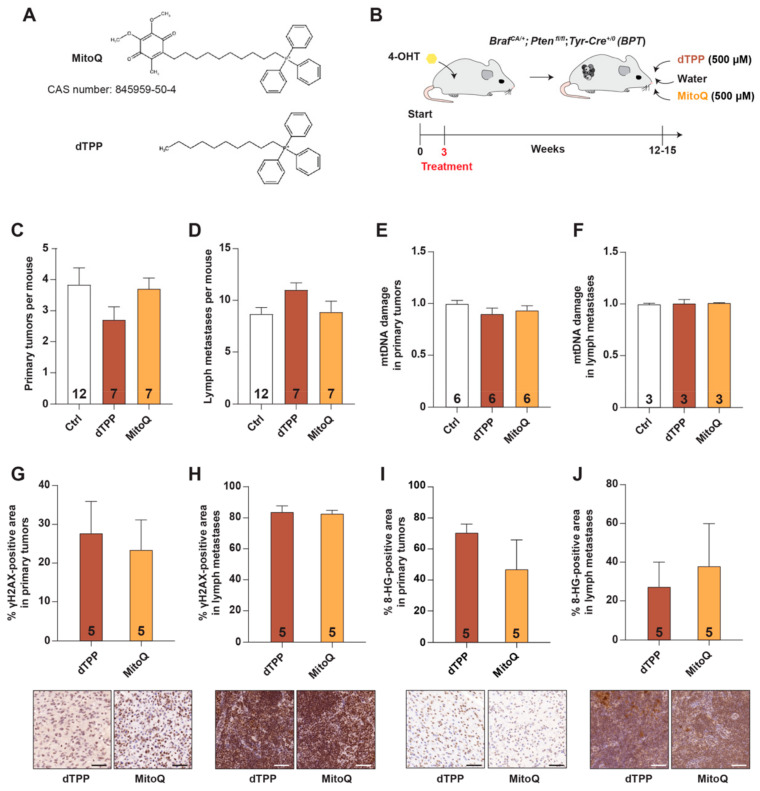
The mitochondria-targeted antioxidant MitoQ does not influence malignant melanoma progression in mice. (**A**) Structure of MitoQ and its control substance dTPP. (**B**) Schematic showing how two-day-old *Braf^CA/+^Pten^fl/fl^Tyr-Cre^+/0^* mice (designated BPT mice) were painted with 4-hydroxytamoxifen on the flank skin to activate BRAF^V600E^ expression and inactivate PTEN expression in melanocytes (start). dTPP and MitoQ were administered in the drinking water to newly-weaned mice (3 weeks). (**C**) Number of primary tumors in BPT mice administered regular water (Ctrl), dTPP, or MitoQ. (**D**) Number of lymph node metastases in BPT mice administered regular water (Ctrl), dTPP, or MitoQ. (**E**,**F**) Quantification of mitochondrial DNA (mtDNA) damage in primary tumors (**E**) and lymph node metastases (**F**); data are normalized to Ctrl. (**G**,**H**) Top, percent H2AX-positive area in primary tumors (**G**) and lymph node metastases (**H**). Bottom, representative photographs of H2AX-stained sections. (**I**,**J**) Top, percent 8-HG-positive area in primary tumors (**I**) and lymph node metastases (**J**) of dTPP- and MitoQ-treated BPT mice. Bottom, representative photographs of 8-HG-stained sections. Numbers in bars = *n*. Scale bars = 50 µm.

To define effects of mitochondria-targeted antioxidants on the progression of malignant melanoma, we first used MitoQ, a MitoQuinone conjugated with the lipophilic triphenylphosphonium (TPP) cation that stimulates its accumulation in the inner mitochondrial membrane [20]. We administered MitoQ and its control compound decyl-TPP (dTPP) in the drinking water of 3-week-old *Braf^CA/+^Pten^fl/fl^Tyr-Cre^0/+^* mice (designated *BPT* mice) [16] with early stages of malignant melanoma (they had been painted with tamoxifen on the flank skin 2–3 days after birth to activate BRAF^V600E^ expression and inactivate *Pten* in melanocytes). The dose and administration route of MitoQ—500 µM in the drinking water—was shown previously to protect against oxidative stress in mice [21,22,23,24,25,26,27] without causing toxicity [18] (Figure 1A,B). MitoQ administration had no significant impact on the number of primary tumors and lymph node metastases in *BPT* mice (Figure 1C,D); and no impact on survival, body weight, lymph metastasis size, and liver and spleen weights (Figure S1A–D). To determine whether MitoQ exerted a protective effect in the mitochondria, we analyzed mitochondrial DNA (mtDNA) damage from primary tumor and lymph node metastasis lysates by PCR. The levels of mtDNA damage in the three groups were similar (Figure 1E,F). Moreover, nuclear DNA damage, as judged by γH2AX and 8-hydroxyguanosine staining, remained unchanged with MitoQ administration (Figure 1G–J).

**Figure 2:**
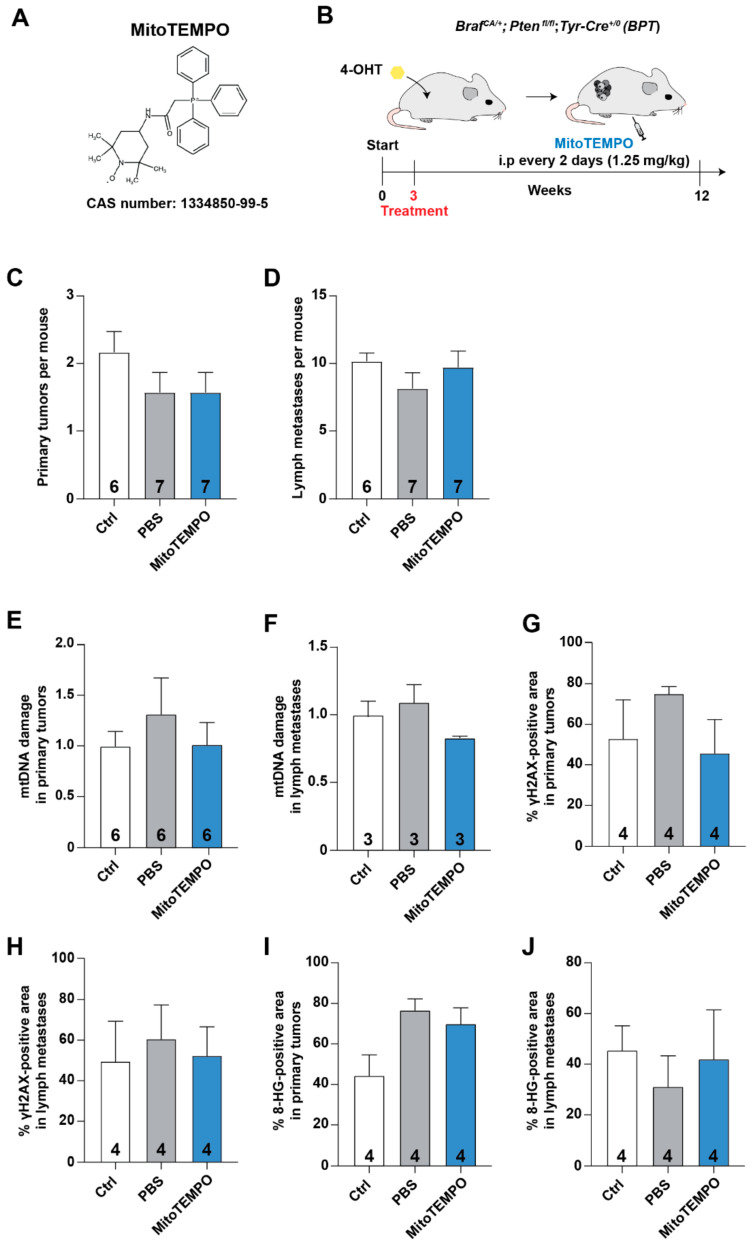
The mitochondria-targeted antioxidant MitoTEMPO does not influence malignant melanoma progression in mice. (**A**) Structure of MitoTEMPO. (**B**) Schematic showing how two-day-old *BPT* mice were painted with 4-hydroxytamoxifen on the flank skin to activate BRAF^600E^ expression and inactivate PTEN expression in melanocytes (start). MitoTEMPO (1.25 mg/kg) or vehicle (PBS) were injected i.p.—starting at weaning (3 weeks)—every other day for a total of 30 injections; a separate group of mice were left untreated (Ctrl). (**C**) Number of primary tumors in Ctrl *BPT* mice and *BPT* mice injected with PBS or MitoTEMPO. (**D**) Number of lymph node metastases in Ctrl *BPT* mice and *BPT* mice injected with PBS or MitoTEMPO. (**E**,**F**) Quantification of mitochondrial DNA (mtDNA) damage in primary tumors (**E**) and lymph node metastases (**F**); data are normalized to Ctrl. (**G**,**H**) Percent γH2AX-positive area in primary tumors (**G**) and lymph node metastases (**H**). (**I**,**J**) Percent 8-HG-positive area in primary tumors (**I**) and lymph node metastases (**J**). Numbers in bars = *n*.

The outcome of the MitoQ melanoma experiments (Figure 1) prompted us to test the effects of MitoTEMPO, a mitochondrial superoxide scavenger, on malignant melanoma progression (Figure 2A,B). Similar to the MitoQ experiments, the dose chosen for the MitoTEMPO experiments was previously reported to protect against oxidative damage without causing toxicity [9,14]. The number of primary tumors and metastases in untreated mice and in mice injected with MitoTEMPO or vehicle did not differ (Figure 2C,D); and the drug did not influence body weight, lymph metastasis size, or liver and spleen weights (Figure S1E–H). Moreover, MitoTEMPO did not influence the levels of mitochondrial and nuclear DNA damage in primary tumors and metastases (Figure 2E–J).

**Figure 3:**
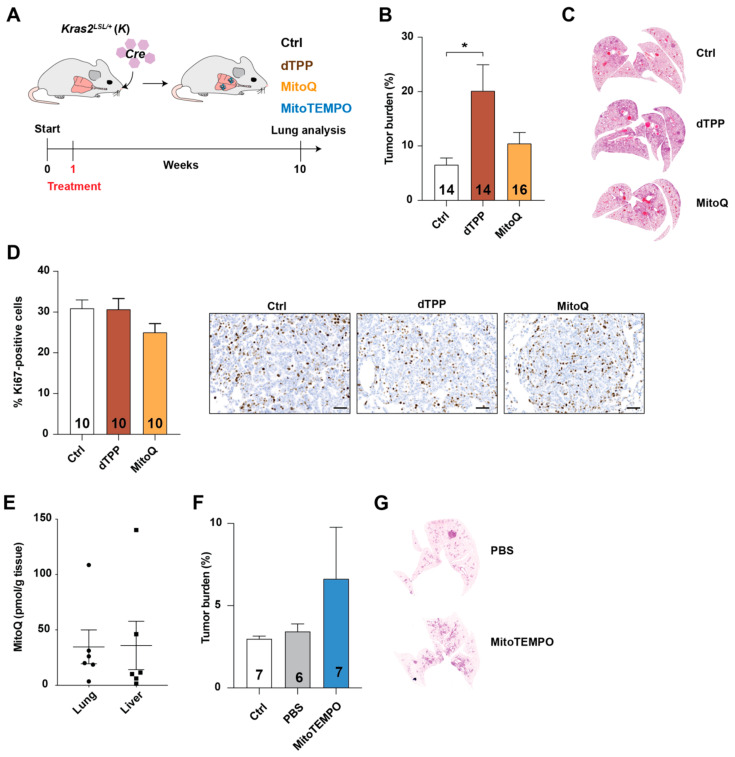
MitoQ and MitoTEMPO does not influence tumor growth in mice with lung cancer. (**A**) Schematic showing how a *Cre*-adenovirus given intranasally to 6–8-week-old *Kras2^LSL/+^* mice activated KRAS^G12D^ expression in the lung (start). One week later, mice were given regular water (Ctrl) or water supplemented with MitoQ (500 µM) or dTPP (500 µM); in separate experiments, mice were left untreated or injected i.p. with MitoTEMPO (1.25 mg/kg) or PBS. (**B**) Tumor burden (percent tumor area per lung area) in lungs from Ctrl, dTPP-treated, and MitoQ–treated *Kras2^LSL/+^* mice, 10 weeks after inhalation of *Cre*-adenovirus. (**C**) Representative hematoxylin and eosin–stained lung sections from mice in panel B. (**D**) Proliferation index (percent Ki67-positive cells). Right panels, representative photographs of Ki67-stained lung sections. (**E**) Mass spectrometry–based detection of MitoQ in lung and liver samples of tumor-bearing *Kras2^LSL/+^* mice administered MitoQ in the drinking water for 10 weeks, as described in panels A and B. (**F**) Tumor burden (percent tumor area per lung area) in lungs from Ctrl and PBS- and MitoTEMPO-injected *Kras2^LSL/+^* mice, 10 weeks after inhalation of *Cre*-adenovirus. (**G**) Representative hematoxylin and eosin–stained lung sections from mice in panel F. Numbers in bars = *n*. Scale bar = 50 µm. * *p* < 0.05.

To investigate the effects of mitochondria-targeted antioxidants on the progression of a different cancer form, we administered MitoQ, dTPP, or no drug, to *Kras2^LSL/+^* mice one week following activation of KRAS^G12D^ expression in the lungs through intranasal inhalation of *Cre*-adenovirus [28] (Figure 3A). Lung tumor burden was similar in MitoQ-treated and untreated mice (Figure 3B,C); oddly however, the mean lung tumor burden was significantly higher in dTPP-treated than untreated mice, and tended to be higher than in MitoQ-treated mice (Figure 3B,C). Nevertheless, the proliferative index in tumors from the three groups of mice did not differ, as judged by Ki67 immunostaining (Figure 3D). MitoQ was detected in lung and liver tissue, as judged by mass spectrometry (Figure 3E). Similar to the results of the MitoQ experiments, MitoTEMPO administration did not significantly affect lung tumor burden (Figure 3F,G).

**Figure 4:**
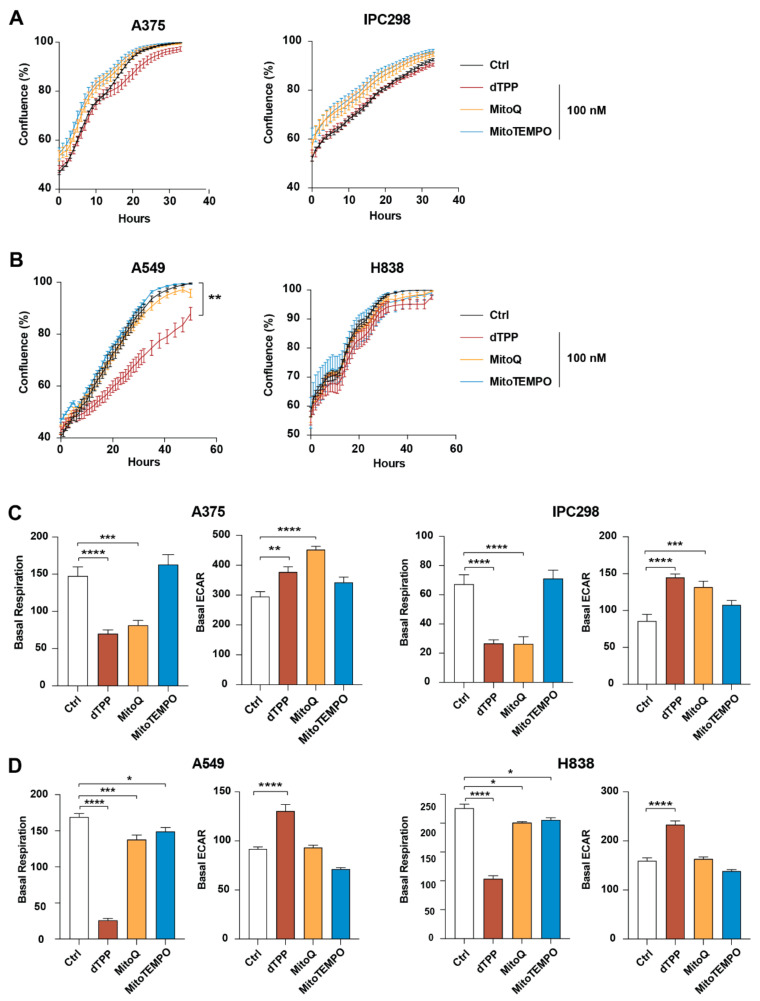
Mitochondria-targeted antioxidants have minimal effects on cell proliferation and inconsistent effects on metabolic parameters in vitro. (**A**) Proliferation of human malignant melanoma cell lines A375 and IPC298 incubated with the indicated compounds in an Incucyte Live-Cell Imaging system. Note *Y*-axis scale. (**B**) Proliferation of human lung cancer cell lines A549 and H838 incubated with the indicated compounds in an Incucyte live-cell imaging system. Note *Y*-axis scale. (**C**) Basal mitochondrial respiration and extracellular acidification rate (ECAR) of A375 and IPC298 melanoma cell lines. (**D**) Basal mitochondrial respiration and extracellular acidification rate (ECAR) of A549 and H838 lung cancer cell lines. * *p* < 0.05; ** *p* < 0.01; *** *p* < 0.001; **** *p* < 0.0001.

We next defined effects of MitoQ and MitoTEMPO on cultured human melanoma and lung cancer cells. The mitochondria-targeted antioxidants did not affect proliferation of the cancer cells; but the control compound dTPP reduced proliferation, particularly in lung cancer cells (Figure 4A,B). To determine whether the mitochondria-targeted antioxidants influence the mitochondrial electron transport chain, we measured the oxygen consumption rate (OCR) and the extracellular acidification rate (ECAR). Both dTPP and MitoQ reduced the basal respiration in melanoma cells, and increased the ECAR, when compared to untreated cells (Figure 4C); MitoTEMPO also increased the ECAR, but did not influence the mitochondrial respiration (Figure 4C). In lung cancer cells, dTPP produced similar effects as in melanoma cells, but MitoQ and MitoTEMPO had little or no discernible effects (Figure 4D). Thus, we find no reproducible effects of MitoQ and MitoTEMPO on cancer cell proliferation and metabolism.

## 4. Discussion

In this study, we found that MitoQ and MitoTEMPO administration had no impact on the number of primary tumors and lymph metastases in mice with BRAF-induced malignant melanoma and no impact on tumor burden in mice with KRAS-induced lung cancer. These results question the rationale of using these mitochondria-targeted antioxidants in anti-cancer therapy.

Even though MitoQ and MitoTEMPO had no impact on malignant melanoma, they actually tended to increase tumor burden compared with untreated mice with KRAS-induced lung cancer. Although this effect was not statistically significant, it is reminiscent of effects of the common antioxidants N-acetylcysteine and Vitamin E, which markedly increased lung cancer progression and metastasis in the same model [17,29]. However, the most marked effect was a statistically significant increase in lung tumor burden in dTPP-treated mice. There is no obvious explanation for this unexpected result, but it demonstrates the importance of using untreated controls in drug experiments; indeed, without the untreated controls, MitoQ would have seemed to reduce lung tumor burden by 50% compared with dTPP treatment.

The mitochondria-targeted antioxidants MitoQ and MitoTEMPO have been successfully used to improve some disease phenotypes of cardiovascular disease [21,27,30,31,32], inflammation [22,23], neurodegenerative disorders [33,34,35,36,37], and diabetes [38,39]. Thus, testing these compounds in other ROS-related disorders such as cancer is highly relevant, particularly as mitochondria-associated ROS are known to stimulate cell proliferation and tumor progression. Indeed, mitochondria-associated ROS can move into the cytosol and subsequently into the extracellular environment, where they can trigger different signaling cascades and pathways [1,3]. Moreover, mitochondrial ROS can target and mutate mitochondrial DNA and thereby contribute to carcinogenesis [10,40]. However, several reports show conflicting results on the efficiency of MitoQ and MitoTEMPO to limit tumor growth and metastasis [9,14,26,41]. Importantly, none of those studies used endogenous mouse models of cancer; and they used either orthotopic injections of melanoma cells [9,14] or chemically-induced hepatocarcinogenesis [26,41].

In our experiments, we used previously established treatment regimens of MitoQ and MitoTEMPO that were proven to suppress oxidative stress in several animal studies [21,22,23,24,25,26,27]. Although we cannot rule out the possibility that using higher doses could produce measurable effects on tumor progression, we were reluctant to use higher doses since this strategy would increase the risk of adverse general effects, as judged by the earlier studies. Moreover, the current doses tended to increase tumor growth, which also makes it unlikely that a higher dose would produce opposite effects that were significant. In line with a previous report [18], mass spectrometry analysis of tissue lysates confirmed the bioavailability of MitoQ, showing that the drug reached the tissue of interest. However, mitochondrial DNA damage remained unchanged across treatments and tissues, suggesting that mitochondria-targeted antioxidants had no protective effect on mitochondrial DNA damage in our studies. Moreover, we observed no differences in ROS-specific and nonspecific DNA damage, as judged by 8-hydroxyguanine and γ-H2AX staining (although these markers might not be able to detect local reduction of ROS and direct DNA damage in the mitochondria). Indeed, the availability of reliable tools to measure endogenous ROS with the sufficient spatial resolution in vivo is still one of the major limitations when studying redox in the biomedical field, although some progress has been recently made [42,43]. Of note, several studies indicate that mitochondria-targeted antioxidants reduce mitochondrial ROS and improve overall mitochondrial fitness [21,22,23,24,25,26,27], whereas others conclude that these compounds could act as pro-oxidants [44,45].

Using the highest dose that did not reduce viability of untransformed cells (fibroblasts, melanocytes), we found that MitoQ and MitoTEMPO did not influence proliferation of human malignant melanoma and lung cancer cells. Importantly, MitoQ increased the ECAR in all human cancer cell lines and markedly reduced the basal mitochondrial respiration in melanoma cells, suggesting that the drug can alter the metabolism of cancer cells. The effect can likely not be attributed to the antioxidant properties of MitoQ since similar results were observed with dTPP. One potential explanation is that accumulation of the cation moiety, which is common to both compounds, in the mitochondrial membrane contributed to the metabolic change. Interestingly, a glycolytic switch has been reported in other studies, where it was also proposed that this family of compounds could create a therapeutic vulnerability to glycolysis inhibitors [13]. The reduced mitochondrial respiration observed with MitoQ has also been linked to protection against ferroptosis [46]; however, that particular study did not use dTPP as control, making it unclear whether the protective effect is due to the quinone or the long alkyl chain. Indeed, several studies also reported deleterious effects of the triphenylphosphonium moiety itself on mitochondrial fitness including autophagy induction, disruption of the ETC, and mitochondrial depolarization [47,48,49,50]. In line with these observations, MitoTEMPO, which does not share the same molecular structure and lacks the long alkyl chain, did not induce metabolic changes.

Overall, our study suggests that the mitochondria-targeted antioxidants MitoQ and MitoTEMPO have little effect as monotherapy agents against malignant melanoma and lung cancer. However, we cannot rule out the possibility that alternative strategies to target mitochondrial ROS might be useful in the clinic, since mitochondria are a major source of cellular ROS [2]. Indeed, given the difficulties linked to the delivery of mitochondria-targeted compounds, there is an increasing need for the generation of endogenous mouse models that can help elucidate the importance of mitochondria-associated ROS in disease [51,52]. Moreover, since we did not observe any effects of these compounds on the tumors and metastases themselves, one possibility would be to explore whether mitochondria-targeted antioxidants instead exert a cytoprotective effect on normal tissues and alleviate off-target effects of standard-of-care treatments [53]. Some studies have also shown advantageous effects when similar antioxidant compounds have been combined with metabolic inhibitors [13], which opens up a possibility for the identification of new metabolic vulnerabilities and potential therapies. Altogether, further studies are needed to clarify the role of mitochondrial redox balance in cancer [54].

In conclusion, although the mitochondria-targeted antioxidants MitoQ and MitoTEMPO have shown promising and exciting results in other pathologies, they do not hinder malignant melanoma and lung cancer progression as monotherapy in our models. Based on these and previous findings [16,17,29,55,56], we warrant caution in the use of antioxidant compounds in cancer.

## Data Availability

Data available on request.

## Supplementary Materials

The following are available online at https://www.mdpi.com/2076-3921/10/2/163/s1, Figure S1. The mitochondria-targeted antioxidants MitoQ and mitoTEMPO do not affect overall health parameters in a mouse model of malignant melanoma.
