# Supplementary material for: Mitochondria-Targeted Antioxidants MitoQ and MitoTEMPO Do Not Influence BRAF-Driven Malignant Melanoma and KRAS-Driven Lung Cancer Progression in Mice
Source: Antioxidants (Basel). 2021 Jan 22;10(2):163. doi: 10.3390/antiox10020163 (PMC7912553; doi:10.3390/antiox10020163)
Supplement: Supplementary file 1 [file antioxidants-10-00163-s001.pdf]

Figure S1

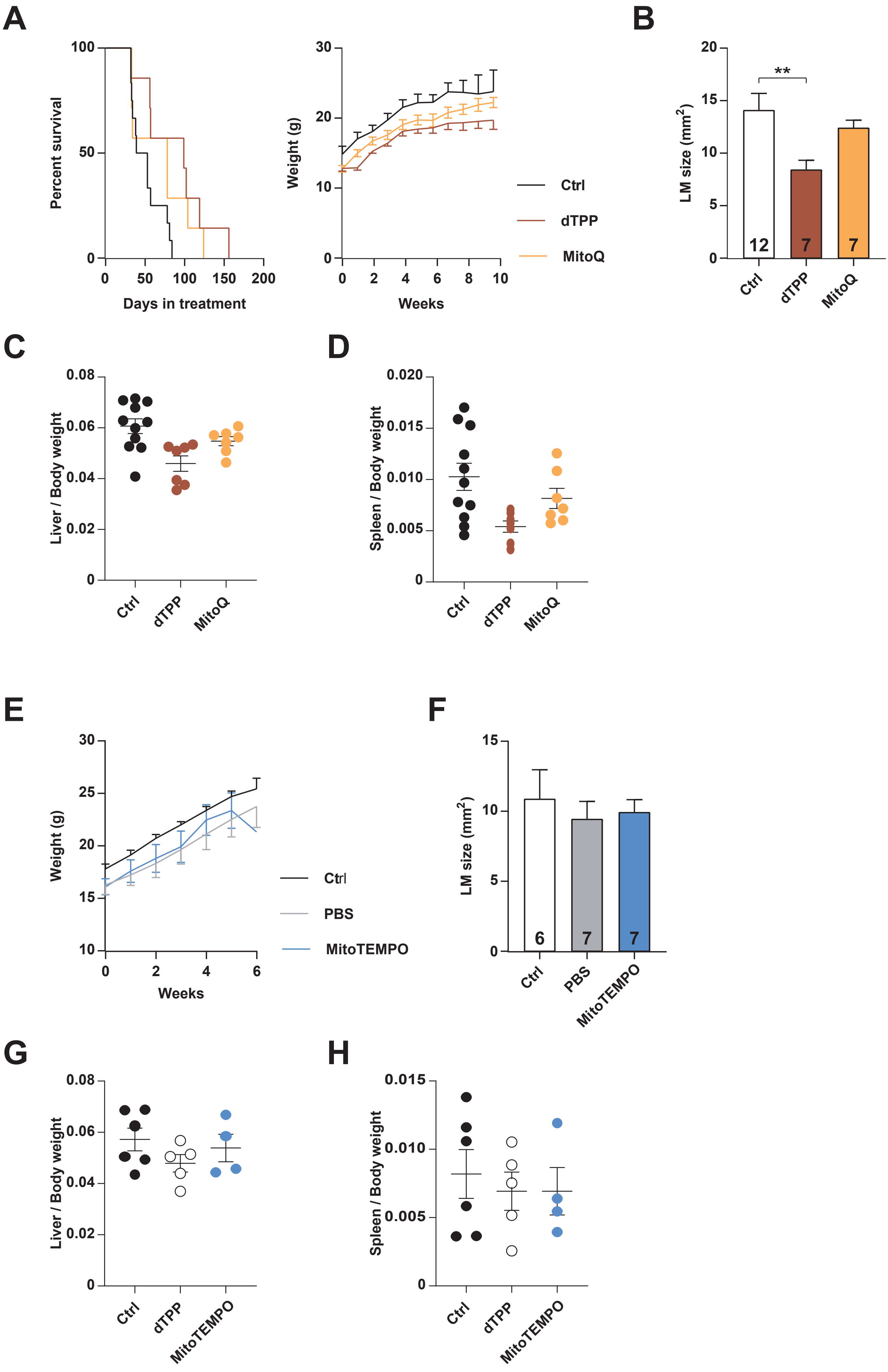

A
Percent survival
100
50
0
0 50 100 150 200
Days in treatment
Weight (g)
30
20
10
0
0 2 4 6 8 10
Weeks
Ctrl
dTPP
MitoQ
B
LM size (mm^2)
20 15 10 5 0
**
12 7 7
Ctrl dTPP MitoQ
C
Liver / Body weight
0.08 0.06 0.04 0.02 0
Ctrl dTPP MitoQ
D
Spleen / Body weight
0.020 0.015 0.010 0.005 0
Ctrl dTPP MitoQ
E
Weight (g)
30 25 20 15 10
0 2 4 6
Weeks
Ctrl
PBS
MitoTEMPO
F
LM size (mm^2)
15 10 5 0
6 7 7
Ctrl PBS MitoTEMPO
G
Liver / Body weight
0.08 0.06 0.04 0.02 0
Ctrl dTPP MitoTEMPO
H
Spleen / Body weight
0.015 0.010 0.005 0
Ctrl dTPP MitoTEMPO

Figure S1. The mitochondria-targeted antioxidants MitoQ and mitoTEMPO do not affect overall health parameters in a mouse model of malignant melanoma. (A) Survival (left) and body weight (right) of BPT mice administered regular water (Ctrl), MitoQ, or dTPP. **(B)** Size of lymph node metastases in BPT mice administered regular water (Ctrl), MitoQ, or dTPP. **(C, D)** Relative liver (C) and spleen (D) weight in BPT mice administered regular water (Ctrl), MitoQ, or dTPP. **(E)** Body weight of untreated BPT mice (Ctrl) and BPT mice administered PBS or mitoTEMPO i.p. (n = 6–7/group). **(F)** Size of lymph node metastases in BPT mice from panel E at the endpoint. **(G, H)** Relative liver (G) and spleen (H) weight of mice in panel E at the endpoint. Numbers in bars = n. ** $P < 0.01$.
